# Gut Microbiota and Developmental Programming of the Brain: From Evidence in Behavioral Endophenotypes to Novel Perspective in Obesity

**DOI:** 10.3389/fcimb.2012.00109

**Published:** 2012-08-15

**Authors:** Melania Manco

**Affiliations:** ^1^Research Unit for Multifactorial Disease, Obesity and Diabetes, Bambino Gesù Children’s HospitalRome, Italy

## Gut Microbiota and Developmental Origin of the Health and Disease

Onset of obesity has been anticipated at earlier ages and prevalence of pediatric obesity has dramatically increased worldwide over the past decades (Han et al., 2010). While epidemic obesity is mainly attributable to westernized lifestyle with excessive consumption of refined carbohydrates and fats and reduced physical activity, onset of obesity in children has been, in part, attributed to the fetus’ exposure to disadvantageous conditions (i.e., hormonal and nutritional cues) during the intrauterine life which can exert a profound impact on the organism’s later development, structure, and function. This phenomenon, which extends to peri- and post-natal periods, is known as “developmental programming of health and disease” (Hochberg et al., 2011).

With the enormous effort that microbiologists are investing in trying to understand the contribution of gut microbiota to human health and disease, a pivotal role of the gut microbiota is emerging as an environmental cue which influences the developmental programming. Most of the evidence has so far been mostly collected with regard to psychopathological “endophenotypes” (i.e., the set of behavioral/physiological symptoms which result into more stable phenotypes of complex traits characterized by low level genetic variability). Epidemiological studies in humans have indicated associations between common neurodevelopmental endophenotypes, such as autism, schizophrenia, depression, and anxiety, with microbial pathogen infections during the perinatal period (Mittal et al., 2008; Finegold et al., 2010). Epidemiological findings have been supported by experimental evidences in germ-free (GF) mice (mice born and raised in a sterile environment and that are devoid of an enteric bacteria) which demonstrated that exposure to microbial pathogens during vulnerable periods result in behavioral abnormalities, including anxiety-like behavior, impaired cognitive function (Sullivan et al., 2006; Goehler et al., 2008), and more elevated home-cage activity counts than conventionalized animals (Bäckhed et al., 2007).

## Paralleling Neurodevelopmental and Obese Endophenotypes. The Brain–Gut–Microbiota Axis

Both neurodevelopmental and obese endophenotypes seem to have their root during the intrauterine life. Early development of gut microbiota may impact the programming of obesity as it does for neurodevelopmental traits.

As observed in mouse models and patients affected by behavioral abnormalities, specific phyla, classes, or species of bacteria, or bacterial metabolic activities characterize also the gut microbiota of obese individuals (Manco et al., 2010). Gut dysbiosis in obesity has been strongly associated with increased ability to harvest energy from the diet, to influence the expression of host genes in particular those regulating lipid and glucose metabolisms at peripheral sites, to promote systemic low-grade inflammation and insulin resistance (Manco et al., 2010). It is, hence, tempting to speculate that gut dysbiosis of individuals prone to obesity may establish in the perinatal life and drive developmental programming of later obesity.

The common soil for development and adult self-sustain of both neurodevelopmental disorders and obesity is the continuous cross-talk between the gut and the brain. The complex bidirectional communication system between gut and brain is vital for maintaining conditions of homeostasis (in these cases, stable behavior, and energy balance, respectively). Through this top-down and bottom-up perspective of information flow, signals from the brain can influence the motor, sensory, and secretory modalities of the gastro-intestinal tract and conversely, visceral messages from the gastro-intestinal trait can influence brain function (Mayer, 2011; O’Mahony et al., 2011). The humans’ internal ecosystem (“human microbiome”) seems to intrude and modify significantly this bidirectional communication starting very early in the life so much to led some to suggest that the brain–gut axis may be more accurately termed “the brain–gut–microbiota axis” (Rhee et al., 2009). Therefore, the human microbiome may enrich and complicate the control system of energy balance which is already one of the most highly integrated and complex functions of the body; not surprisingly, given its importance. At the level of the central nervous system, information from the periphery is integrated and allows initiating appropriate behavioral, humoral, and neural outputs which are often common to both endophenotypes.

Recognition of the interaction between gut microbiota and central nervous system may shed new light to explain epidemic obesity (Forsythe et al., 2010; Bercik et al., 2011; Cryan and O’Mahony, 2011) by explain some of its trans-generational transmission.

## Gut Colonization, Programming of the Hypothalamic-Pituitary-Adrenal, and Stress Response

Microbial colonization of mammals is an evolution-driven process that modulates many host physiological functions, many of which are associated with nutrient intake. Colonization of the infant gut commences at birth when delivery exposes to a complex microflora. The infant’s microbiota expresses unquestionably a maternal fingerprint, but soon after the birth, the newborn organism is rapidly, and densely populated with complex forms of indigenous microbes (Manco et al., 2010; Putignani et al., 2010).

Gut colonization has been found to exert an effect on the development of the hypothalamic-pituitary-adrenal (HPA) axis and on the acute response to stress conditions (Sudo et al., 2004). Dysregulation of the HPA axis and impaired stress response are common to different behavioral endophenotypes such as depression, anxiety, but also visceral obesity. Indeed, impaired stress response influences several metabolic pathways, including some involved in the control of satiation, body growth gluconeogenesis, insulin resistance, and insulin secretion (Chrousos, 2000). GF mice exhibit a less anxious phenotype in the elevated plus maze, a well validated model of anxiolytic action (Sudo et al., 2004; Neufeld et al., 2011) and an exaggerated release of corticosterone and adrenocorticotrophin hormone (ACTH) compared to the specific pathogen free (SPF) animals in response to a mild restraint stress induced (Sudo et al., 2004). Administration of exogenous glucocorticoids (the equivalent of corticosterone in mice) is known to reduce synaptophysin expression in the fetal brain of non-human primates (Antonow-Schlorke et al., 2003). Therefore, excessive release of steroids during vulnerable periods of the life can be one of the mechanisms by which gut microbiota modulates HPA neuroplasticity and may, hence, enhance the risk to develop obesity later in adulthood.

In experiments comparing the stress response of GF and SPF, a decrease in the brain derived neurotrophic factor (BDNF) was reported (Sudo et al., 2004). BDNF is a key neurotrophin involved in neuronal growth and survival, which regulates the growth and differentiation of new neurons and synapses; it is involved in the regulation of multiple aspects of cognitive and emotional behaviors (Zola et al., 2000). BDNF is involved in the regulation of appetite and control of energy metabolism (Rothman et al., 2012). There was also a decreased expression of the *N*-methyl-d-aspartate (NMDA) receptor subunit 2a (NR2a) in the cortex and hippocampus of GF animals compared to SPF controls (Sudo et al., 2004). More recently, Neufeld et al. (2011) reported, in contrary, an up-regulation of the BDNF expression in the dentate gyrus of the hippocampus of GF animals. The researchers found also a decrease in the NR2B subunit of the NMDA receptor in the amygdala of GF animals.

In obesity, which is often characterized by impaired hippocampal synaptic plasticity and cognitive abilities such as learning and memory, significant decreases in NR2A and NR2B subunit expressions have been observed in the hippocampus of obese animals and their expressions seem to significantly increase in the obese rats following 60% calorie restriction (Yilmaz et al., 2011).

### Modulation of microbiota and brain reprogramming

The response to stress stimuli observed in the GF mice is partially reversed by re-colonization with fecal matter from SPF animals and fully restored by mono-association with *Bacillus infantis* in a time dependent manner (Sudo et al., 2004).

Studies investigating the effect of administration of probiotics (i.e., live microorganisms which when administered in adequate amounts confer a health benefit on the host) support a role for the microbiota in anxiety-like behaviors but with divergent effects depending on the strain.

For example, administration of *Lactobacillus helveticus* R0052 and *Bacillus longum* R0175 taken in combination, induced anxiety-like activity in rats (Messaoudi et al., 2011), while chronic treatment with *Lactobacillus rhamnous* (JB-1) over 28 days produced animals with lower levels of stress induced corticosterone release and reduced depressive behaviors in the forced swim test in addition to a less anxious phenotype in the elevated plus maze. Animals treated by *L. rhamnosus* also showed alterations of gamma-aminobutyric acid (GABA)B1b mRNA in the brain with increased expression in the hippocampus, amygdala, and locus coeruleus as reduced GABAAα2 mRNA expression in prefrontal cortex and amygdala and increased GABAAα2 in the hippocampus. Interestingly, the authors demonstrated that vagotomized mice did not display the neurochemical and behavioral effects caused by the *L. rhamnosus*, thus implicating the vagus nerve in the direct communication between the bacteria and the brain (Bravo et al., 2011). As to commonalities between pathogenesis of behavioral disorders and obesity, there are two remarks. The increased expression of GABAAα2 gene in the brainstem, hippocampus, and amygdala is described in the Prader–Willi syndrome, a form of genetic obesity, characterized by compulsive food seeking behaviors (Scoles et al., 2011). As to the nerve vague, it plays a pivotal role in the gut-brain axis control of food intake (Sam et al., 2012). However, the anti-anxiety effect of the *L. rhamnosus* (*Lr* JB-1) via the vagus nerve and the central GABA system observed in healthy mice by Bravo et al. (2011), confirmed previous results by Bercik et al. (2011) who, however, observed that this effect is independent of the vagus nerve.

### Gut Microbiota and release of brain transmitters

Gut microbiota influences the release of some of the major brain transmitters which act in the gut-brain axis and modulate food intake and energy balance (Gruninger et al., 2007), i.e., short chain fatty acids (SCFAs), Peptide YY (PYY), tryptophan, serotonin, endocannabinoid ligands, and ghrelin. For instance, the interaction between SCFAs produced by the gut bacteria, and Gpr41 increases circulating levels of PYY, a potent orexigenic agent (Samuel et al., 2008). Conventionalized GF mice present with a 2.8-fold increase in plasma serotonin levels respect to control animals (Wikoff et al., 2009). Administration of *B. infantis* 35624 to Sprague-Dawley rats, for example, has been shown to induce an elevation in plasma tryptophan levels, a precursor to serotonin (Desbonnet et al., 2008). Diet supplementation with prebiotic fiber has been associated with alterations in the expression or content of various gut hormones linked to the regulation of energy balance, notably increasing the satiety hormone PYY and reducing the expression of the orexigenic peptide ghrelin (Delzenne et al., 2005).

## Conclusion and Future Perspectives

In conclusion, as learned by experiments on acute response to stress in GF and SPF mice, gut microbiota initiates a signaling soon after birth at a time when the newborn mice become exposed to gut microbiota.

Alternatively, it is tempting to speculate that exposure to gut microbiota metabolites, generated by the flora of the pregnant mother, can influence brain development during embryogenesis. Hence the influence of the maternal gut microbiota may contribute to the trans-generational transmission of endophenotypes characterized by dysfunctional HPA, primarily neurodevelopmental and obese endophenotypes. So, investigation of the impact of gut microbiota on the developmental programming of obesity and modulation of adult gut microbiota (“re-programming”) by different microbial strains represent research priorities. Such investigation will reveal the full therapeutic potential of nurturing gut bacteria starting since the perinatal life to prevent obesity and associated comorbidities and to reduce the burden of obesity by modulating not only energy harvesting, but influencing feeding behaviors and energy expenditure. Mechanisms of action which deserve investigations include release and turnover of neurotransmitters, altered parasympathetic activity and expression profiles of canonical signaling pathways. Thus, probiotic, prebiotic, or antimicrobial administration and the evaluation of food-related behaviors and metabolic outputs, in healthy and obese humans, are worthwhile pursuits in order to reduce the burden of epidemic obesity.
